# EHMTI-0381. Visual images can prove to be an important tool to aid in the diagnosis of cluster headaches

**DOI:** 10.1186/1129-2377-15-S1-I1

**Published:** 2014-09-18

**Authors:** I Khan, H Zafar, F Ahmed

**Affiliations:** 1 Neurology, Hull Royal Infirmary, Hull, UK

## Background

Cluster headache is one of the most distressing and painful clinical conditions seen in medical practice, and affect 1-2 % of the population. Although its clinical presentation does not vary widely in terms of the symptoms experienced by patients, it still presents a significant diagnostic challenge particularly to the primary and secondary care physicians. This results in patients having to bear with agonizing symptoms before they are given the correct diagnosis and appropriate therapy. The time taken to diagnose cluster headache has reduced from 22 years in the 1970’s to 3.4 years about 8 years ago1 and on average a patient sees 5 doctors before receiving the correct diagnosis., however, the severity of the condition merits early recognition.

## Aims and objectives

To evaluate the use of visual images and verbal description of pain as an aid to diagnosis in primary and secondary care but not limited to them.

## Method

12 cluster headache patients were interviewed along with 10 migraine patients used as a control (as it is the most common misdiagnosis). The questions were aimed at identifying patients’ symptoms with a set of visual images which they were shown and their verbal description of pain. The diagnosis of cluster headache and migraine was based on the ICHD-III beta criteria from the International Headache Society.

## Results

The description of pain remained consistent for cluster headache patients due to the excruciating nature of the pain whereas it varied in migraineurs depending on the individual pain threshold of patients. Patients with cluster headache chose visual images different to migraineurs. We conclude that visual images are an efficient way to describe symptoms in cluster headache patients and could be used as a vital tool to aid diagnosis.

No conflict of interest.

